# HIV-Associated Anal Cancer

**DOI:** 10.7759/cureus.14834

**Published:** 2021-05-04

**Authors:** Pushti Khandwala, Sachi Singhal, Devashish Desai, Meghana Parsi, Rashmika Potdar

**Affiliations:** 1 Internal Medicine, Crozer Keystone Health System, Upland, USA; 2 Internal Medicine, Crozer Keystone Heath System, Upland, USA; 3 Hematology and Medical Oncology, Crozer Keystone Health System, Upland, USA

**Keywords:** anal cancer, hiv, hpv, hpv vaccine

## Abstract

Anal cancer, despite being a rare malignancy, is increasing in incidence, accounting for 0.5% of all new cancer cases in the United States, with rate of new cases being 1.9 per 100,000 men and women. It is common in immunocompromised individuals, especially those with malignancy, human immunodeficiency virus (HIV) and human papillomavirus (HPV) infection. Despite similar treatment of anal cancer in both HIV-positive and negative patients, guidelines for prevention and treatment of therapy-related side effects are rarely studied. While these patients have a better prognosis on HAART, limited guidelines exist regarding appropriate therapy. There is a common link between HPV and HIV and the transmission of one is associated with increased risk of transmission of the other. HPV vaccine which is known to prevent high-grade cervical intraepithelial neoplasia is thought to also decrease the incidence of anal intraepithelial neoplasia. The association of HPV vaccine in the prevention of anal cancer in high-risk groups with HIV is a scarcely studied subject that requires further research.

## Introduction and background

Anal cancer is a rare malignancy, accounting for 0.5% of all new cancer cases in the United States, with rate of new cases being 1.9 per 100,000 men and women [1]. According to the American Cancer Society statistics, it is estimated that there will be about 8,590 new anal cancer cases (5,900 in women and 2,690 in men) and about 1,350 deaths (810 in women and 540 in men), accounting for 0.2% of all cancer deaths.

The incidence of anal cancer has been steadily rising over the years, between 2001 and 2015 anal cancer cases rose by 2.7% each year [2]. With the advances in treatment, the five-year relative survival rate is 68.7%.

Major risk factors associated with an increased incidence of anal cancer include female gender, human papillomavirus infection (HPV), human immunodeficiency virus infection (HIV), multiple sexual partners, receptive anal intercourse, history of cervical, vulvar or vaginal cancer, immunosuppression associated with solid organ transplantation, hematological malignancy, autoimmune disease and smoking [3-8].

With the rise of HIV, HPV infections, and anal cancer, in this article, we aim to provide a comprehensive review of their associations, current treatment modalities and highlight areas for future research.

## Review

Pathology

The anal canal ranges from the perianal skin, or anal verge, to the rectal mucosa. Squamous mucosa lines the anal canal from the distal-most anal verge up to the dentate or the pectinate line, following which is a transition zone consisting of both squamous and nonsquamous (either urothelium-like transitional or rectal glandular) mucosa [9]. Tumors arising within the anal canal distal to the dentate line are most often keratinizing squamous-cell carcinomas whereas those appearing in the transitional mucosa above the dentate line are frequently nonkeratinizing squamous-cell carcinomas. On the other hand, adenocarcinomas act differently and are grouped with rectal carcinomas. The transformation zone (TZ) in the anal canal is the area where the columnar epithelium of the rectum changes into the nonkeratinizing squamous epithelium of the anus; this is similar to the transformation zone (or squamocolumnar junction) in the cervix. It is thought that basal layer cells in the epithelium of the TZ can become infected with HPV after micro-abrasions. There are over 40 sexually transmitted types of HPV that infect the skin and mucous membranes. In infected individuals, the immune system normally eliminates the virus within two years, but in some individuals, it becomes persistent [10]. Persistent infections can give cause to dysplasia. Increasing severity of anal intraepithelial neoplasia (AIN) is graded as AIN-1, AIN-2, and AIN-3. Recently AIN-1 is often referred to as low-grade AIN (LGAIN) and AIN-2, and AIN-3 together as high-grade AIN (HGAIN). HGAIN is regarded as a precursor for anal squamous cell carcinoma (SCC). It is known that each type of HPV has a varied capacity for inducing malignant change. Low-risk HPV types are associated with low-grade dysplastic changes, whereas the high-risk HPV types which are: 16 and 18, types 31, 33, 35, 39, 45, 51, 52, 56, 58, 59, 68, 73, and 82 are more identified in lesions associated with high-grade dysplasia or invasive carcinoma [11]. In a study done by Frisch et al., high-risk types of HPV, notably HPV-16, were detected in 84% of the anal-cancer specimens examined [12]. Another similar study, wherein 282 specimens were tested, high-risk HPV was found in 88% [13].

Associations between HPV, HIV and anal cancer

Anal cancer incidence rates are 30 times higher in HIV-infected individuals compared to the general population [14]. In a large North American cohort study conducted by Silverberg et al. [5], anal cancer incidence rates among 34,189 HIV-infected (55% MSM, 19% other men, 26% women) and 114, 260 HIV-noninfected individuals (90% men) were compared. The unadjusted anal cancer incidence rates per 100,000 person-years were found to be 131 for HIV-infected MSM, 46 for other HIV-infected men, and 2 for HIV-noninfected men. HIV-infected women had an anal cancer rate of 30/100 000 person-years, and no cases were observed for HIV-noninfected women. The widespread availability of highly active antiretroviral therapy (HAART) was thought to change the landscape. However, a large French cohort study done by Piketty et al. [15] which looked at temporal trends, showed an increased incidence of anal cancer between the pre-HAART and HAART era that plateaued thereafter. It was seen that anal cancer occurred earlier in HIV-infected patients than in the general population. Even in HIV patients with a CD4 cell count greater than 500/μL for more than two years before diagnosis, the risk was more than 20-fold higher. Anal infections with high-risk HPV (hrHPV) are very common among HIV-infected men who have sex with men (MSM) [16,17]. The relationship between HIV, HPV and anal cancer is complex. Both HPV and HIV are sexually transmitted and transmission of one is associated with an increased risk of transmission of the other. In addition to immunosuppression associated with HIV, transmission of HPV could be associated with increased persistence of HPV and decreased clearance.

The correlation between HIV and anal cancer is difficult to understand, due to the confounding factors. A systematic review of 95 studies was done by Lin et al., which included studies published from January 1986 to July 2017 in MEDLINE, Embase, and the Cochrane Library on anal HPV infection [18]. It showed that a steady rise in HPV-16 positivity was associated with increasing severity of diagnoses from normal anal cytology to high-grade lesions and to anal cancer, irrespective of sex and HIV status. HIV was shown to influence the natural history of HPV types, with the result that the fraction of anal cancer attributable to HPV-16 was lower in HIV-positive than HIV-negative anal cancer.

Treatment

Historically, anal cancer was treated by abdominoperineal resection (APR) with permanent colostomy. This changed when a landmark trial by Nigro et al. proved the success of radiotherapy (RT) with chemotherapy alone in complete pathological cure [19]. The medications mitomycin-C (MMC) and 5-fluorouracil (5-FU) have become the standard of care ever since. Combined mitomycin-5-FU therapy slows colostomy rates and higher disease-free survival throughout several trials [20]. The combination of chemotherapy with RT has proven to be superior to either alone. The current standard of care followed by clinicians worldwide is concurrent 5-FU, MMC and RT. In HIV-positive patients, the same combination treatment is used as the standard of care. When patients have been on HAART, treatment outcomes are comparable to HIV-negative population, albeit with a higher risk of acute events. These acute events have not been shown to impact long-term outcomes in any manner. Most studies found the HIV-positive patients with anal cancer to be more likely to be a younger male black [21-23]. Most of them had received HAART in the six months before cancer treatment. These studies demonstrated a higher risk of adverse events in the HIV-positive population, with grade 3 or 4 hematological toxic effects in particular [22]. Grade 3/4 acute skin events were also reported. However, this did not translate into poorer treatment outcomes as both groups had similar survival and -ostomy placement rates. The CD4 count is another major prognostic factor in outcomes. Hoffman et al. demonstrated in 17 patients that pretreatment counts >200 correlated with excellent disease control on chemoradiation with acceptable morbidity, versus patients with <200 CD count who showed markedly increased morbidity [24]. Edelman et al. further confirmed the significance of CD counts predicting outcomes when 7/8 patients with CD4 count >200 survived at 18 months versus 4/7 in the low count arm [25].

The widespread use of antiretrovirals started around 1999-2000. These drugs control HIV proliferation and amplification. Along with a high CD4 count, being on HAART leads to favorable outcomes in HIV patients. Stadler et al. compared pre-HAART group of HIV-positive individuals receiving therapy for anal cancer with those receiving HAART and reported a marked difference in their 24-month survival- 17% for pre-HAART group versus 67% for those on HAART. The mean CD counts were 190 and 255, respectively, in the pre-HAART and HAART treatment groups. Of note, all six patients in the pre-HAART group passed away with SCC versus only two in the HAART group. Four out of six remaining in the HAART group were disease-free on all follow-up visits [26]. Other studies also showed independent association of overall survival with high viral loads and low CD4 counts [27]. All these studies are limited by a small cohort size and retrospective nature of these studies. Almost all studies demonstrated higher toxicities in HIV-positive patients.

Of note, an appropriate choice of antiretrovirals is also imperative. Recommended initial regimens include two nucleoside reverse transcriptase inhibitor (NRTIs; abacavir/lamivudine or tenofovir disoproxil fumarate/emtricitabine) and a third single/boosted drug which is either an integrase strand transfer inhibitor (dolutegravir, elvitegravir or raltegravir), a non-nucleoside reverse transcriptase inhibitor (NNRTI; efavirenz or rilpivirine) or a boosted protease inhibitor (PI; darunavir or atazanavir). As per the 2014 recommendations of the International Antiviral Society-USA panel, dolutegravir- or raltegravir-based regimens are to be used in the setting of anticancer and immunosuppressive drug use [28]. Zidovudine is generally avoided due to its side effect profile- it causes nausea, anemia and myelosuppression, all of which are worsened by chemotherapy [29].

Table 1 illustrates several trials which studied the effects of different treatment modalities in patients with HIV and anal cancer.

**Table 1:** Treatment outcomes/toxicities and adverse events in patients with HIV being treated for anal cancer. RT: radiotherapy; 5-FU: 5-fluorouracil; MMC; mitomycin-C; ADRs: adverse drug reactions; AEs: adverse events; OS: overall survival.

Study	Bryant et al. (2018) [21]	Grew et al. (2015) [30]	Bongrand et al. (2011) [23]	Seo et al. (2009) [31]	Janne et al. (2008) [22]
Mean age	55 vs 63 years	52 vs 64 years	46 vs 62 years	N/A	48 vs 62 years
Other notable characteristics	31.3% vs 9.2% blacks	Higher rates of hospitalization in HIV+ (33 vs 15%)	95% vs 23% men	No correlation found between CD counts and degree of acute toxicities and OS	93% vs 25% males
Mean pre-treatment disease status	Pre Rx CD4: 370/uL	N/A	Pre Rx viral load: <200 copies/mL in 75% pts	N/A	N/A
Treatment	Intensity-modulated radiotherapy, mitomycin C chemotherapy	Chemoradiation	Chemoradiotherapy with 60 Gy pelvic irradiation, cisplatin-based chemotherapy, surgery for local failures/ complications	RT with 5-FU and MMC	RT alone or with chemotherapy: 5-FU and MMC/cisplatin
ADRs					
3/4 Hematological AEs	58.7% vs 39.7%	N/A	N/A	N/A	33% vs 12%
Any treatment toxic effects	36.2 vs 26.3%	N/A	N/A	N/A	3/4 skin AEs: 35% vs 17%
Outcomes					
All-cause ostomy	14% vs 13% at 5 years	N/A	N/A	N/A	N/A
All-cause mortality	13% vs 19%	N/A	N/A	N/A	N/A
OS	N/A	42% vs 67% at 3 years	39% vs 84% at 5 years	91.7 vs 83.6% at 3 years	61% vs 65% at 5 years
Local disease control	N/A	N/A	50% vs 77%	N/A	38% vs 87%

Management of adverse drug reactions

Major toxicities resulting from the treatment of anal cancer are diarrhea, skin desquamation and immunosuppression. HIV-positive patients receiving treatment for anal cancer experience higher toxicities and require more frequent and longer treatment breaks than their HIV-negative counterparts. This effect is further exacerbated by lower CD counts and untreated HIV [24]. Dermal and hematological adverse events are seen significantly more than in HIV-negative groups. HIV-positive patients undergoing treatment were more frequently hospitalized within 90 days of treatment for acute toxic effects, in particular, for hematological toxicities [21]. Chemotherapy also exacerbates a neutropenia that already exists in HIV/AIDS patients, which can be ameliorated by granulocyte colony-stimulating factors (G-CSF) [32]. However, these drugs are not to be administered concurrently with chemoradiotherapy [33]. Patients may also need antibiotics as treatment and/or prophylaxis due to the increased risk of opportunistic infections (OI). As there are no clear guidelines in place for management of chemotherapy-related toxicities in HIV-positive patients, both HIV-related and cancer-related guidelines need to be reviewed and considered while managing these patients.

HPV vaccine in anal cancer

The causal relationship between HPV infection and anal cancer is well established [34]. The quadrivalent HPV (qHPV) vaccine is efficacious in preventing persistent cervical infection with HPV-6, 11, 16, or 18 and the associated high-grade CIN. However, its effectiveness was not clear on preventing AIN. Palefsky et al. conducted a large double-blind study in 2011, wherein 602 healthy men who have sex with men, 16 to 26 years of age were randomly assigned to receive either qHPV or placebo. There was an effectiveness of 50.3% against HPV-6, 11, 16, or 18 (95% CI 25.7 to 67.2) in the intention-to-treat population and 77.5% (95% CI 39.6 to 93.3) in the per-protocol population. In the intention-to-treat group, the rates of AIN per 100 person-years were 17.5 and 13 in the placebo group and vaccine group, respectively. Furthermore, in the per-protocol group, rates were 8.9 and 4 in the placebo and vaccine group, respectively. The rate of grade 2 or 3 AIN related to infection with HPV-6, 11, 16, or 18 was reduced by 54.2% (95% CI 18.0 to 75.3) in the intention-to-treat population and by 74.9% (95% CI 8.8 to 95.4) in the per-protocol efficacy population [35]. This clearly demonstrates the role of qHPV vaccine in the prevention of anal cancer by decreasing the incidence of the precursor lesions. The effect of this vaccine on incidence of anal cancer, however, remains to be studied. Since it has reduced the rate of intraepithelial neoplasia, it will most likely reduce the incidence of anal cancer. Effectiveness of this vaccine in HIV-positive patients is another area that needs further research.

## Conclusions

Anal cancer is common in HIV-positive patients. Treatment in HIV-positive patients is similar to HIV-negative patients but is associated with increased toxicity and needs careful monitoring. Patients on HAART have a better prognosis compared to those not on HAART. Effect of HPV vaccine on incidence of anal cancer and interaction with HIV needs further research.
